# Subcortical dopamine D_2_ receptor availability and glucose metabolism in autism: a dual-tracer PET/MR study

**DOI:** 10.1007/s00259-026-08053-4

**Published:** 2026-07-06

**Authors:** Laust Vind Knudsen, Manouchehr Seyedi Vafaee, Ziba Ahangarani Farahani, Abigail Jane Sheldrick-Michel, Tanja Maria Michel

**Affiliations:** 1 Psychiatric Research Unit, Odense University Hospital, Odense, Denmark; 2 University of Southern Denmark, Odense, Denmark; 3 Department of Nuclear Medicine, Odense University Hospital, 5000 Odense, Denmark

**Keywords:** PET, MRI, Glucose metabolism, Dopamine, Autism, Functional connectivity

## Abstract

**Purpose:**

Dopaminergic signalling and glucose metabolism have been implicated in autism spectrum disorder (ASD), yet in vivo evidence in the human brain remains largely unexplored. This study examined subcortical dopamine D_2_ receptor availability and glucose metabolism in ASD to assess their clinical relevance.

**Methods:**

In this dual-tracer PET/MR case–control study, 30 autistic and 30 neurotypical adults (age-, sex-, body mass index-, and IQ-matched) underwent [^11^C]raclopride PET to assess dopamine D_2_ receptor availability, [^1^⁸F]FDG PET to measure glucose metabolism and resting-state fMRI to evaluate functional connectivity.

**Results:**

Autistic individuals demonstrated increased D_2_ receptor availability in the thalamus, with additional increases in the nucleus accumbens and putamen among autistic males compared to neurotypical males. Glucose metabolism was elevated in the thalamus and globus pallidus in ASD relative to NT, and this pattern persisted within both autistic males and autistic females in sex‑stratified comparisons. Globus pallidus and thalamic glucose metabolism correlated positively with social and communication difficulties. Resting-state fMRI analyses revealed diagnosis- and sex-dependent correlations between thalamic D_2_ receptor availability and functional connectivity.

**Conclusions:**

This study provides in vivo evidence of elevated subcortical D_2_R availability and glucose metabolism in autistic adults, a pattern observed across sexes. We also show that D_2_R availability is tightly linked to glucose metabolism and that D_2_R-functional connectivity coupling is altered in ASD, both relative to neurotypical adults and between autistic males and females. These findings highlight dopaminergic mechanisms as potential biomarkers and therapeutic targets in ASD.

**Supplementary Information:**

The online version contains supplementary material available at https://doi.org/10.1007/s00259-026-08053-4.

## Introduction

Autism spectrum disorder (ASD) is a neurodevelopmental condition affecting approximately 2% of adults [1], characterized by differences in social communication, repetitive behaviours, and sensory processing [2]. Existing pharmacological interventions primarily target associated symptoms such as irritability, while core characteristics, remain insufficiently addressed. Moreover, diagnosis continues to rely on behavioural assessments, underscoring the need for objective neurobiological markers and novel molecular targets.

Growing evidence implicates dysregulation of the dopamine system, particularly involving D_2_ receptors (D_2_R), in the neurobiology of ASD [3]. This hypothesis is supported by the fact that the only FDA-approved medications for ASD (risperidone and aripiprazole) act on the D_2_Rs as an antagonist and a partial agonist, respectively [4]. These medicaments are used to treat non-core ASD symptoms such as agitation and irritability, which affects approximately 90% of autistic children [5]. Moreover, preclinical studies have demonstrated that modulation of dopaminergic pathways can induce or alleviate autism-like behaviours [6, 7]. Likewise, genetic associations with the dopamine transporter gene (DAT1) [8], increased striatal D_2_R mRNA expression in postmortem tissue [9], and the consistent finding that diverse ASD mouse models exhibit increased D_2_R density in the nucleus accumbens (Nacc) [10] further implicate dopaminergic dysregulation as a promising mechanistic feature of ASD.

However, in vivo investigations of D_2_R availability (i.e., the availability of receptors for radioligand binding, which reflects receptor density and/or competition from endogenous dopamine) in autistic individuals remain limited. Only three positron emission tomography (PET) studies have examined D_2_R availability in ASD, reporting reduced thalamic [11] and striatal [12] D_2_Rs availability in ASD, as well as diminished phasic striatal dopamine release during an incentive task [13]. Nevertheless, no study has investigated how D_2_R availability relates to large-scale functional organization or cerebral metabolism. Given the high concentration of D_2_Rs in striatal and subcortical regions [14], and the strong influence of D_2_R availability on functional connectivity (FC) [15] and cerebral metabolism [16], a multimodal resting-state approach is essential for a more comprehensive characterization of dopaminergic abnormalities in ASD. Sex differences represent an additional critical gap. The dopamine system exhibits marked sex‑specific variations in D_2_R availability [17], and ASD presents differently across sexes, where females are often underdiagnosed [18]. Although sex differences in FC have been demonstrated in ASD [19], no PET study in ASD has examined sex differences.

To address these gaps, we conducted a dual‑tracer PET/MR study in autistic and neurotypical (NT) adults with balanced sex representation. Using [^11^C]raclopride to quantify D_2_R availability, [^1^⁸F]FDG to assess glucose metabolism, and resting‑state fMRI to evaluate connectivity, we aimed to provide the first integrated characterization of dopaminergic and metabolic alterations in ASD across diagnostic groups and sexes.

## Methods and materials

### Participants

Adults (age ≥ 20 years) with full‐scale IQ > 80 and capacity to consent were enrolled in this case–control study. Autistic participants were referred by the diagnostic team in Odense or recruited via advertisements; all held a prior clinical diagnosis of ASD according to DSM-5 criteria [2]. NT adults were recruited through advertisement. Recruitment was stratified to maintain a 1:1 male–female ratio within each diagnostic group, despite ASD being more prevalent in males [1], in order to ensure balanced representation across sexes.

All participants completed the Autism‑Spectrum Quotient (AQ) [20] and the Edinburgh Handedness Inventory [21] on the scanning day, prior to PET acquisition. Cognitive ability was extracted from prior Wechsler Adult Intelligence Scale (WAIS) or Wechsler Intelligence Scale for Children (WISC) assessments; individuals without an existing measure completed a WAIS‑IV screening on the scanning day (Supplementary Methods).

Exclusion criteria included MRI contraindications, neurological disorders, nicotine or stimulant use, overnight fasting noncompliance (verified by blood glucose), and significant MRI abnormalities. Five ASD participants on stable medication adhered to standardized washout or continuation rules (details in Supplementary Methods). None of the medications directly targeted the dopaminergic system. Demographic and clinical characteristics are summarized in Table 1.

**Table 1:** Participant information

Characteristic	ASD (*n* = 30)		NT (*n* = 30)		Analysis		
	N	%	N	%	X^2^	df	p
Female	15	50	15	50	0	1	1.0
Race							
White	28	93.33	27	90	0.22	1	0.640
Black	1	3.33	1	3.33	0	1	1
Asian	1	3.33	2	6.67	0.35	1	0.550
	Mean	SD	Mean	SD	t	df	p
Age (years)	24.59	3.14	25.99	3.26	1.69	58	0.096
BMI	23.72	4.25	25.24	4.15	1.41	58	0.165
Laterality quotient	75.33	46.29	81.33	38.57	0.54	58	0.588
PET dose							
[^11^C]Raclopride (MBq)	426.46	53.08	425.48	42.07	0.08	58	0.939
[^18^F]FDG (MBq)	297.81	48.15	308.83	38.92	0.81	58	0.409
Blood glucose (mmol/L)	4.97	0.33	5.07	0.51	0.94	58	0.350
Intelligence quotient	105.6	12.39	107.4	8.96	0.63	56	0.529
AQ							
Total score	32.57	5.69	12.87	5.79	13.3	58	< 0.001
Social skills	6.43	2.28	1.67	1.88	8.82	58	< 0.001
Attention switching	7.60	1.96	3.40	2.31	7.59	58	< 0.001
Attention to detail	6.50	1.66	3.07	1.62	8.13	58	< 0.001
Communication	6.73	2.08	2.30	1.82	8.77	58	< 0.001
Imagination	5.30	2.15	2.43	1.68	5.76	58	< 0.001

*AQ* Autism Spectrum Quotient; *BMI* Body mass index; *MBq* megabecquerel; *Mmol/L* Millimoles Per Liter; *SD* standard deviation

### PET and MRI acquisition

Full acquisition parameters and details are provided in Supplementary Methods. Briefly, imaging was performed on a 3 T integrated SIGNA PET/MR scanner (GE Healthcare) with a 19‑channel head‑and‑neck coil between November 2022 and October 2024. Participants underwent a 60‑minute [^11^C]raclopride bolus PET scan (mean dose: 425.98 ± 47.55 megabecquerel [MBq]) at 11.17 AM (± 11.16 min). After five [^11^C] half‑lives (~ 102 min), a 60‑minute [^1^⁸F]FDG scan was acquired (mean dose: 303.32 ± 48.41 MBq). Simultaneous structural MRI (T1/T2) and resting‑state fMRI were collected during the [^11^C]raclopride PET scan.

### PET and MRI preprocessing

Full descriptions of all preprocessing pipelines, quality-control procedures, and software versions are provided in Supplementary Methods. PET data were processed using the MAGIA pipeline [22] in MATLAB (version R2021B). Motion correction and anatomical registration were performed in SPM12 (Wellcome Centre for Human Neuroimaging, London, UK; http://www.fil.ion.ucl.ac.uk/spm). [^1^⁸F]FDG Standardized uptake value ratio (SUVR) was computed using data from the 30–60 min post-injection interval, with cerebellar grey matter as the reference region. Given that prior ASD [^1^⁸F]FDG‑PET studies have used varying reference regions [23], we validated this choice in our dataset, confirming no group differences in cerebellar grey matter tracer uptake (Supplementary Fig. S1). [^11^C]Raclopride non-displaceable binding potential (BPnd) was estimated using the simplified reference tissue model with cerebellar grey matter as reference region [24]. Resting‑state fMRI was processed using the CONN toolbox (version 22; default preprocessing and denoising steps) [25]. Spatial normalization, smoothing, and confound regression followed standard procedures. All acquisition and preprocessing details, including motion thresholds, and analytic parameters, are provided in Supplementary Methods.

### Statistical analysis

All analytic details, including software packages, permutation settings, and model specifications, are fully described in Supplementary Methods. Group differences in demographics used t‑tests or χ^2^ tests as appropriate.

We prioritized voxelwise analyses because D_2_R density varies substantially across subcortical nuclei [26], whereas region-of-interest (ROI)-based averaging can conceal this spatial heterogeneity and thereby mask localized effects. Voxelwise comparisons of [^11^C]raclopride BPnd and [^1^⁸F]FDG-SUVR were conducted using FMRIB’s Software Library (FSL, version 6.0.7.10) nonparametric permutation testing with 5,000 permutations and threshold‑free cluster enhancement (TFCE) [27], controlling family-wise error (FWE) at p-FWE < 0.05. Parametric BPnd values were not thresholded, and negative values were retained. Analyses were restricted to an a priori bilateral subcortical mask (caudate, Nacc, putamen, globus pallidus (GP), thalamus; Harvard–Oxford atlas, 50% threshold; 5,307 voxels) [28]. Regions were selected based on prior evidence of reliable, robust [^11^C]raclopride binding in these regions [17, 26]. Covariates were age and sex; BMI was additionally included for [^1^⁸F]FDG due to its influence on cerebral glucose metabolism [29]. Secondary contrasts assessed sex differences within ASD and NT groups, and group‑by‑sex interactions, motivated by known sex effects in D_2_R availability [17], glucose metabolism [30], and FC [31, 32].

Complementary ROI‑based analyses were performed. Nonparametric permutation analysis of covariance (20,000 permutations) tested group and sex effects on ROI measures and their correlation with AQ scores, with false‑discovery‑rate (FDR) correction [33]. Full results are provided in the Supplementary Materials. VoxelStats [34] was used to assess voxelwise correlations between [^11^C]raclopride BPnd and [^1^⁸F]FDG-SUVR corrected for multiple comparisons (p < 0.001 voxel-level; cluster-level p-FWE < 0.05). Multivariate pattern analysis (MVPA) in CONN evaluated associations between thalamic BPnd and voxelwise whole‑brain FC patterns (i.e., voxel-to-voxel FC), followed by seed‑to‑voxel post‑hoc tests (p < 0.001 voxel-level; p < 0.05 cluster-level FDR correction [33]. Additional details are in Supplementary Methods.

## Results

Thirty autistic and 30 NT participants completed the imaging protocol (Supplementary Fig. S2). Groups did not differ in demographics, scanner‑related variables, or IQ, but autistic participants scored higher on all AQ subscales (Table 1).

### Group differences in [^11^C]raclopride BPnd

**Fig. 1:**
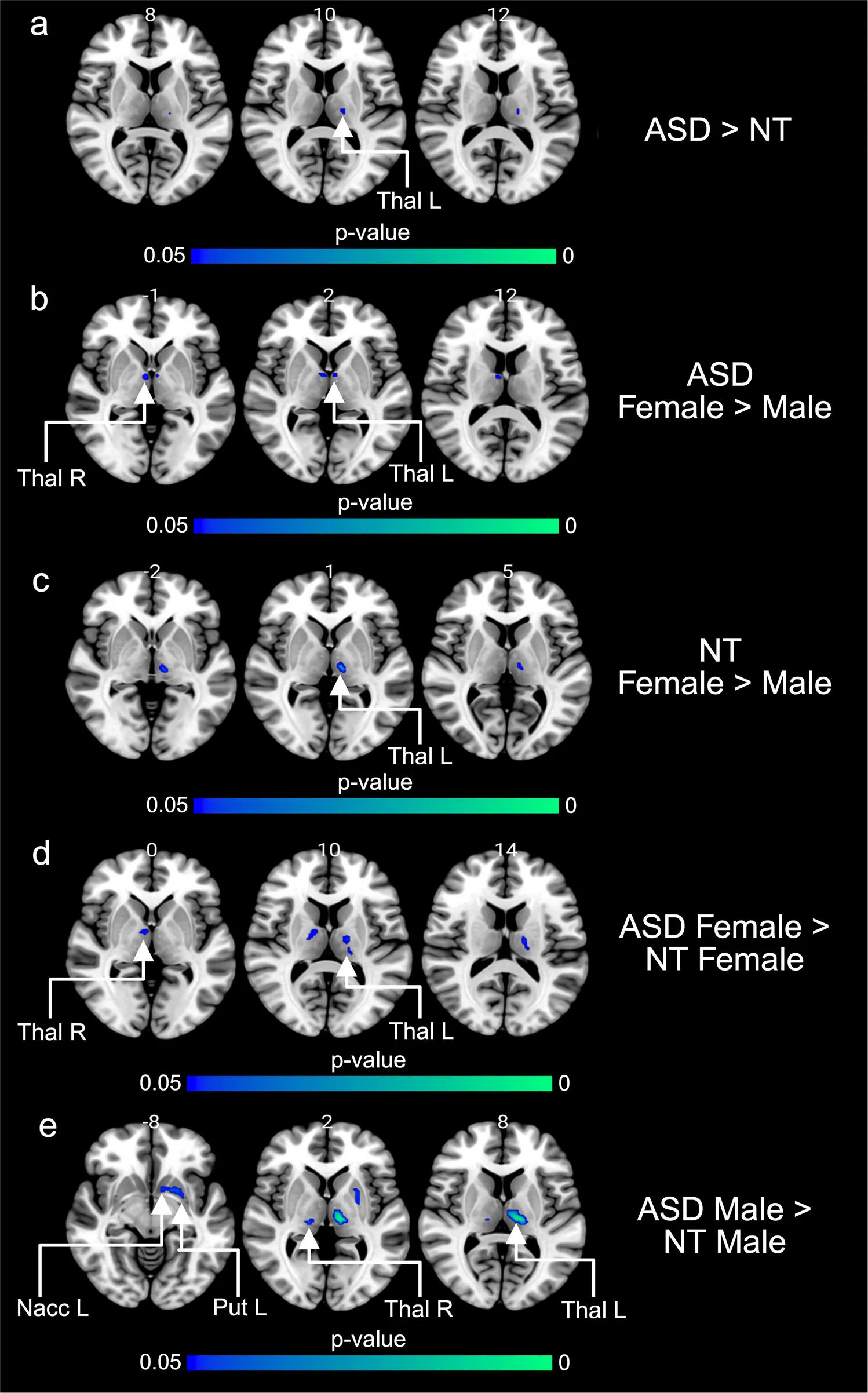
Voxelwise comparisons of dopamine D_2_ receptor availability using [^11^C]raclopride PET. **A**) Individuals with ASD show higher [.^11^C]raclopride non-displaceable binding potential (BPnd) compared to NT (*n* = 60). Panel **B**-**C**: Sex effects within diagnostic groups: females exhibit greater BPnd than males within both ASD B) (*n* = 30) and NT C) (*n* = 30) groups. Group-by-sex comparisons show elevated BPnd in ASD females **D**) and ASD males **E**) relative to same-sex NT controls (*n* = 30). Colour bars represent voxelwise *p*-values, thresholded at p < 0.05, family-wise error (FWE) corrected using Threshold-Free Cluster Enhancement (TFCE). Axial slices were selected to highlight peak voxels in each contrast. Thalamus (Thal), putamen (Put), and nucleus accumbens (Nacc)

The voxelwise analysis revealed a small cluster of increased left‑thalamic [^11^C]raclopride BPnd in ASD compared with NT (9 voxels, 72 mm^3^, the highest uncorrected t-value (peak t) = 3.36, degrees of freedom (df) = 56, and the lowest FWE-corrected p-value (peak p-FWE) = 0.044, Montreal Neurological Institute (MNI) coordinates: –16, –19, 10) (Fig. 1A). A similar trend was observed in the ROI analysis (Supplementary Table S1).

### Sex differences in [^11^C]raclopride BPnd

Autistic females demonstrated higher bilateral thalamic [^11^C]raclopride BPnd compared to autistic males (58 voxels, 464 mm^3^, peak t = 3.60, df = 27, peak p-FWE = 0.039, MNI coordinates: 4, –6, -2) (Fig. 1B). Congruently, the comparison between NT males and females demonstrated enhanced left thalamic D_2_R availability in NT females (51 voxels, 408 mm^3^, peak t = 4.81, df = 27, peak p-FWE = 0.018, MNI coordinates: -8, –18, 0) (Fig. 1C).

Next, we compared the diagnostic groups, beginning with females, demonstrating significantly higher bilateral thalamic D_2_R availability in autistic females compared with NT females (103 voxels, 824 mm^3^, peak t = 3.59, df = 27, peak p-FWE = 0.032, MNI coordinates: -8, –18, 0) (Fig. 1D). Similarly, autistic males showed significantly higher D_2_R availability than NT males in the bilateral thalamus, left Nacc and right putamen (434 voxels, 3472 mm^3^, peak t = 4.89, df = 27, peak p-FWE = 0.004, MNI coordinates: -10, -20, 2) (Fig. 1E). ROI-based results exhibited similar trends in general albeit not reaching statistical significance (Supplementary Table S2 and Supplementary Fig. S3).

### Group differences in [^18^F]FDG-SUVR

**Fig. 2:**
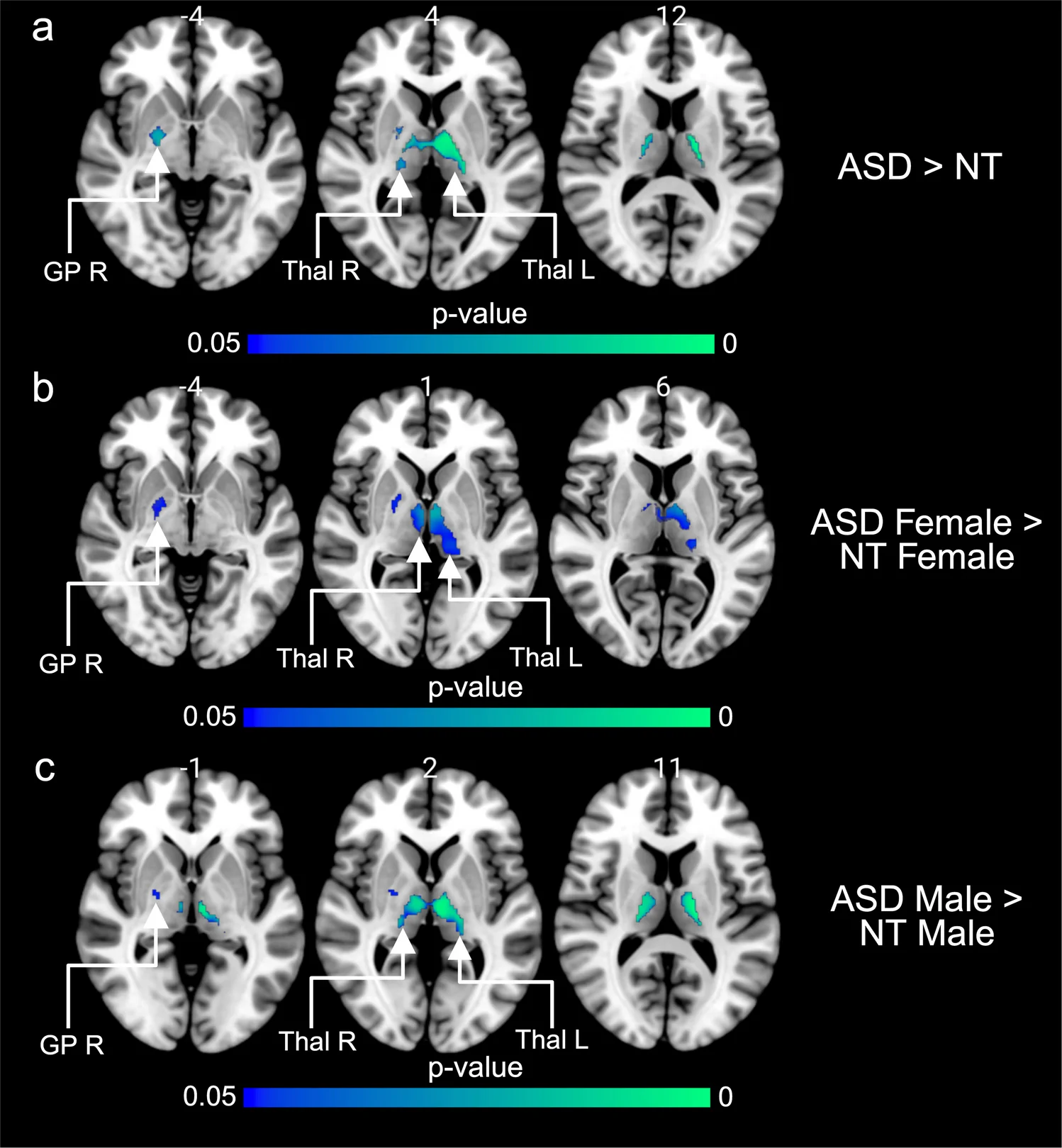
Voxelwise group comparison of glucose metabolism using [^18^F]FDG PET. **A**) Autistic individuals demonstrated significantly higher [^1^⁸F]Fluorodeoxyglucose ([^1^⁸F]FDG) uptake compared to NTs (*n* = 60). **B**) ASD females exhibit greater [^1^⁸F]FDG uptake than NT females (n = 30). **C**) ASD males exhibit greater [^1^⁸F]FDG uptake than NT males (*n* = 30). Clusters are displayed on axial slices selected to highlight peak voxel locations for each contrast. Colour bars indicate p-values. All results were thresholded at *p* < 0.05, family-wise error (FWE) corrected. Anatomical regions with significant clusters are labelled: globus pallidus (GP) and thalamus (Thal). R = right; L = left

The voxelwise [^18^F]FDG-SUVR analysis demonstrated significantly higher glucose metabolism in the bilateral thalamus and right GP in ASD compared with NT (825 voxels, 6600 mm^3^, peak t = 4.06, df = 55, peak p-FWE = 0.004, MNI coordinates: -18, –16, 11) (Fig. 2A). ROI analyses supported these findings (Supplementary Table S1).

### Sex differences in [^18^F]FDG-SUVR

No significant differences in glucose metabolism were evident between autistic males and females (peak p-FWE = 0.281) or between NT males and females (peak p-FWE = 0.224). However, autistic females showed significantly higher [^18^F]FDG-SUVR than NT females in the right GP and bilateral thalamus (825 voxels, 6600 mm^3^, peak t = 3.62, df = 26, peak p-FWE = 0.017, MNI coordinates: -2, –8, -2) (Fig. 2B). Although not reaching significance similar trends were observed in the ROI-based analysis (Supplementary Table S2 and Supplementary Fig. S4). Congruently, autistic males demonstrated significantly higher [^18^F]FDG-SUVR than NT males in the right GP and bilateral thalamus (4533 voxels, 36264 mm^3^, peak t = 4.52, df = 26, peak p-FWE = 0.005, MNI coordinates: -18, -18, 10) (Fig. 2C).

### Voxelwise correlation between [^1^⁸F]FDG-SUVR and [^11^C]raclopride BPnd

**Fig. 3:**
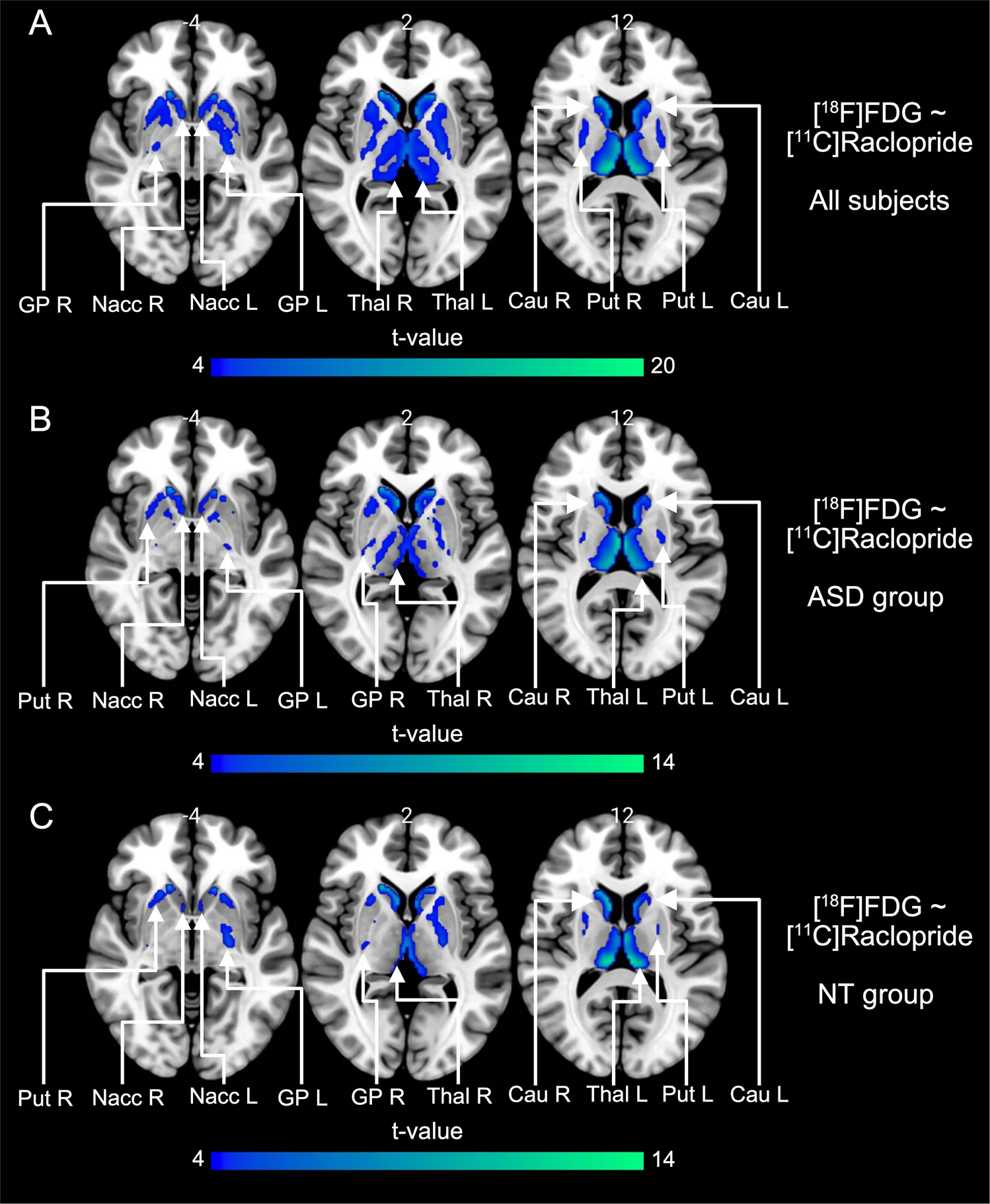
Voxelwise correlation between [^1^⁸F]FDG-SUVR and [^11^C]raclopride BPnd. **A**) Voxelwise positive correlation between [^1^⁸F]FDG-SUVR and [^11^C]raclopride non-displaceable binding potential (BPnd) across the full sample (n = 60). **B**) Voxelwise correlation between [^1^⁸F]FDG-SUVR and [^11^C]raclopride BPnd in the ASD group (*n* = 30) and **C**) NT group (*n* = 30). Clusters are displayed on axial slices selected to highlight peak voxel locations for each contrast

Voxelwise analysis revealed a significant positive correlation, linking increased D_2_R availability to higher glucose metabolism across the bilateral caudate, GP, Nacc, thalamus, and putamen (3,948 voxels, 31,584 mm^3^, df = 56, peak t-FWE = 20.84, MNI coordinates: -4, -10, 16) (Fig. 3A). Because both D_2_R availability and glucose metabolism were elevated in ASD, we repeated the correlations within each group to rule out between‑group confounding. Both autistic (Fig. 3B) and NT participants (Fig. 3C) still showed significant positive correlation between D_2_R availability and glucose metabolism across the caudate, GP, Nacc, thalamus, and putamen (ASD: 2,746 voxels, 21,968 mm^3^, df = 26, peak t-FWE = 17.09, MNI coordinates: 6, -15, 18. NT: 2,116 voxels, 16,928 mm^3^, df = 26, peak t-FWE = 15.04, MNI coordinates: 10, 14, 14).

### Correlation between PET and AQ score

**Fig. 4:**
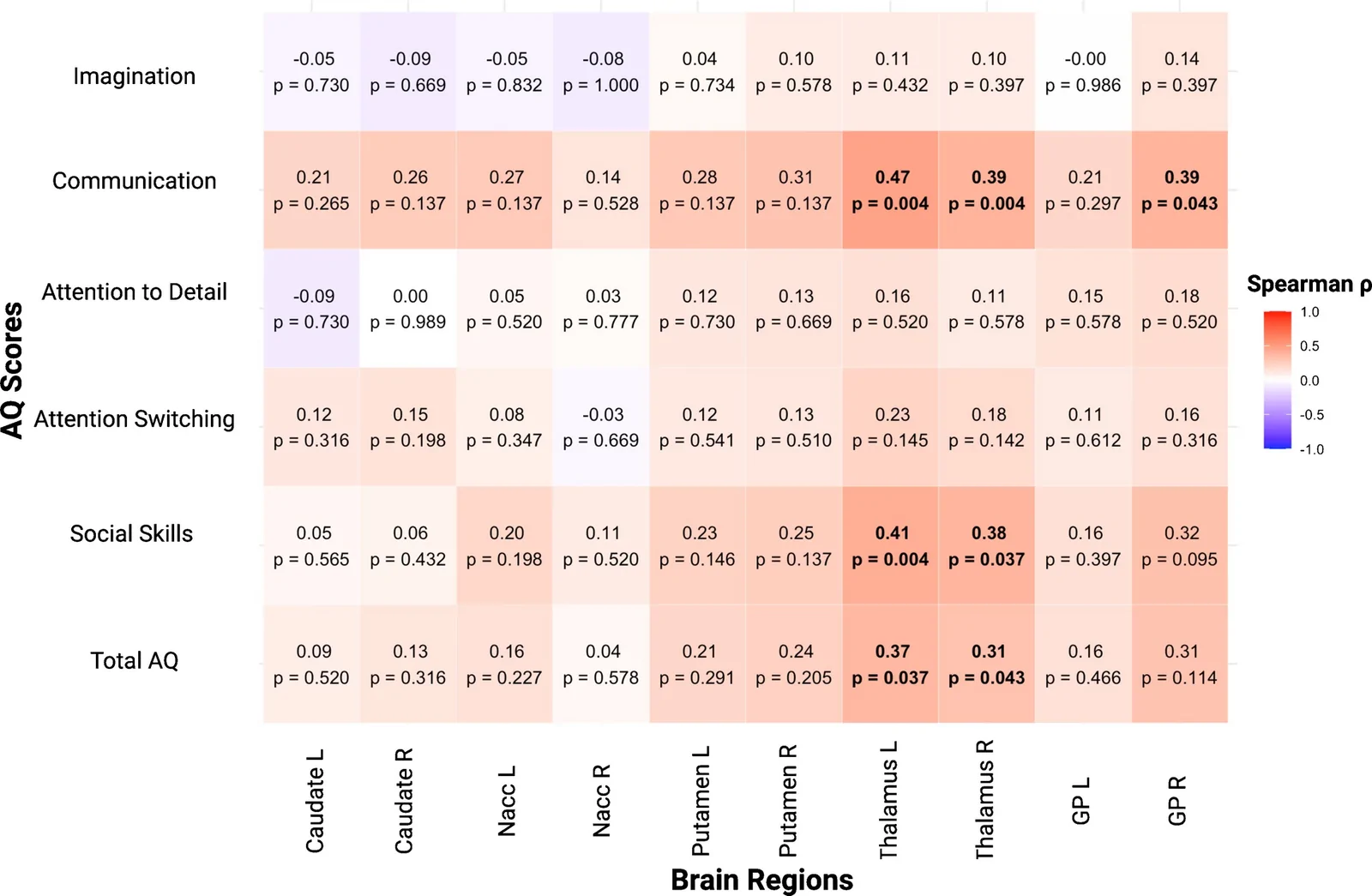
Correlations Between [^1^⁸F]FDG-SUVR and Autism Spectrum Quotient scores. Heatmap showing Spearman correlations (ρ) between regional [^1^⁸F]FDG standardized uptake value ratios (SUVRs) and AQ subscale and total scores across striatal and subcortical regions. Statistically significant correlations (*p*-FDR < 0.05) are highlighted in bold

We also performed a correlations analysis between AQ scores and regional glucose metabolism (Fig. 4). [^1^⁸F]FDG-SUVR was positively correlated with AQ communication scores in the left thalamus (r = 0.474, df = 55, p-uncorrected = 0.0002, p-FDR = 0.004), right thalamus (r = 0.394, df = 55, p-uncorrected = 0.0006, p-FDR = 0.004), and right GP (r = 0.390, df = 55, p-uncorrected = 0.005, p-FDR = 0.043). Similarly, positive correlations between glucose metabolism and AQ social skills were observed in the left thalamus (r = 0.413, df = 55, p-uncorrected = 0.001, p-FDR = 0.004) and right thalamus (r = 0.384, df = 55, p-uncorrected = 0.004, p-FDR = 0.038). AQ total scores were also positively correlated with glucose metabolism in the left (r = 0.366, df = 55, p-uncorrected = 0.006, p-FDR = 0.037) and right thalamus (r = 0.314, df = 55, p-uncorrected = 0.008, p-FDR = 0.043). No significant correlations were found between AQ scores and [^11^C]raclopride BPnd.

### Resting-state fMRI

**Fig. 5:**
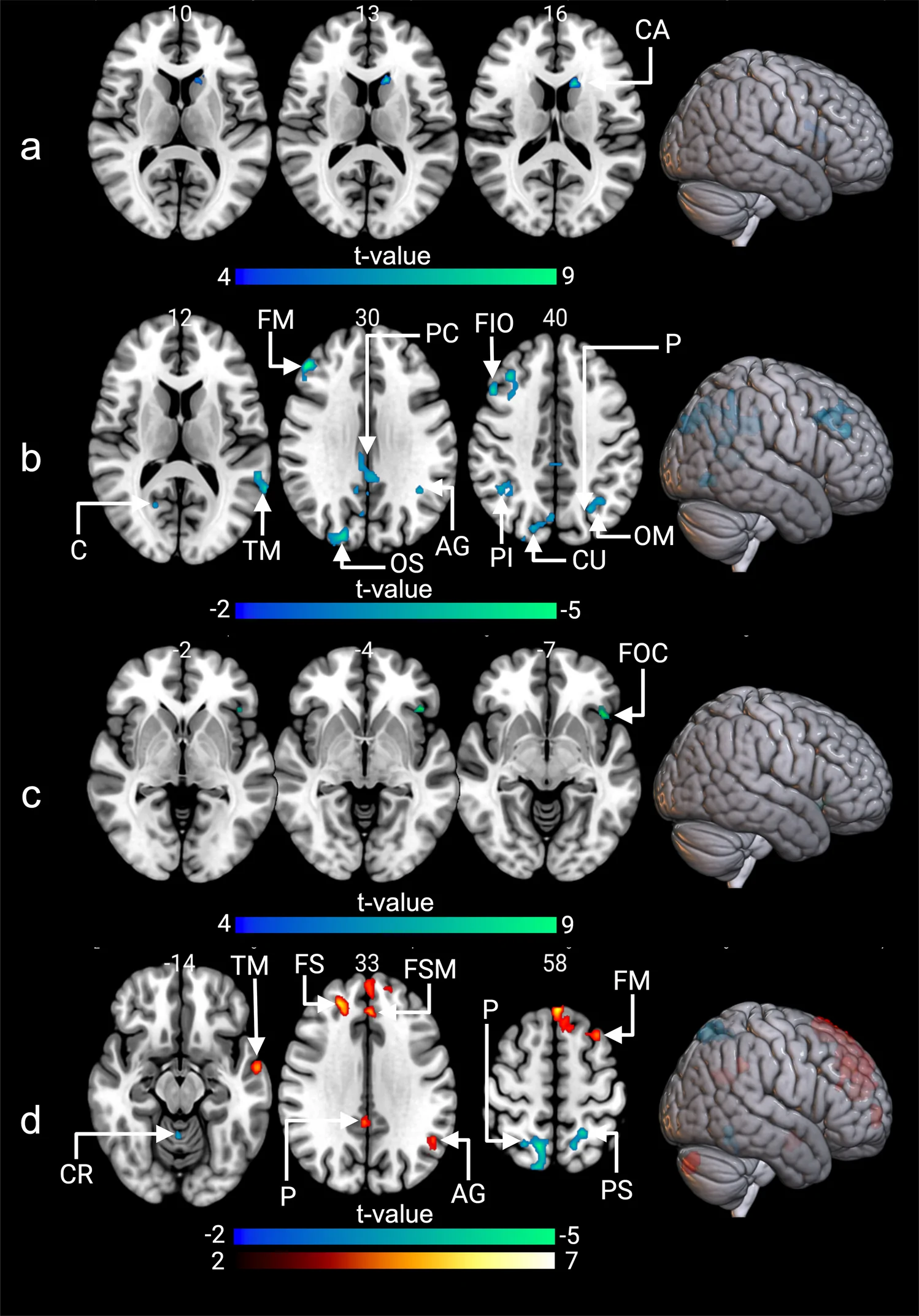
T-statistic maps from resting-state fMRI analyses assessing the correlation between functional connectivity and dopamine D_2_ receptor availability. **A**) Multivariate pattern analysis (MVPA) identified a significant cluster in the left thalamus showing group differences in the correlation between FC and [^11^C]raclopride non-displaceable binding potential (D_2_R availability) in ASD compared to NT participants. **B**) Post hoc seed-to-voxel analysis revealed a brain-wide negative correlation between left thalamic D2R availability and FC originating from the left caudate (CA) in ASD. **C**) MVPA comparing ASD males and ASD females showed significant sex-related patterns. **D**) Post hoc seed-to-voxel analysis using the frontal orbital cortex seed demonstrated sex differences in the correlation between left thalamic D_2_R availability and FC. Anatomical abbreviations: AG = angular gyrus, C = calcarine, CA = caudate, CR = cerebellum, CU = cuneus, FIO = frontal inferior operculum, FM = frontal middle gyrus, FOC = frontal orbital cortex, FS = frontal superior gyrus, FSM = frontal superior medial gyrus, OM = occipital middle gyrus, OS = occipital superior gyrus, P = precuneus, PC = posterior cingulate, PI = inferior parietal lobule, PS = superior parietal lobule, TM = temporal middle gyrus

Given the elevated left thalamic D_2_R availability in the ASD group (Fig. 1A), and in autistic females (Fig. 1D) and males (Fig. 1E) compared with their NT counterparts, we used the left thalamic BPnd as an independent variable in the MVPA to examine its correlation with whole-brain FC. This analysis identified a significant cluster in the left caudate (Fig. 5A, df = 56, p-uncorrected =  < 0.00001, p-FDR = 0.0004, MNI coordinates: –14, 18, 14, 134 voxels), suggesting that left thalamic [^11^C]raclopride BPnd affects left caudate FC differently in ASD compared with the NT group. Post hoc seed-to-voxel analysis from the left caudate cluster revealed eight significant clusters, the largest in the right cuneus (Fig. 5B, df = 56, p-uncorrected =  < 0.00001, p-FDR = 0.00001, MNI coordinates: 18, –78, 36, 371 voxels). In all clusters, stronger negative correlations between left thalamic BPnd and left caudate FC were observed in ASD compared with NT (Supplementary Table S3A). Thereby suggesting that, higher left thalamic D_2_R availability was associated with greater reductions in caudate–cortical connectivity in ASD.

We further explored whether the relationship between left thalamic BPnd and FC differed by sex within the ASD group. A significant cluster emerged in the left frontal orbital cortex (Fig. 5C, df = 26, p-uncorrected = 0.0002, p-FDR = 0.005, MNI coordinates: -46 18 -10, 54 voxels), suggesting that the correlation between left thalamic BPnd and FC differs between autistic males and females. Post hoc seed-to-voxel analysis revealed ten significant clusters, the largest in the left superior frontal gyrus (Fig. 5D, df = 26, p-uncorrected =  < 0.00001, p-FDR =  < 0.0001, MNI coordinates: -2, 18, 56, 1492 voxels). Full cluster statistics are reported in Supplementary Table S3B. No significant correlation between D_2_R availability and FC were found between NT males and females (peak p-FDR = 0.183).

## Discussion

To our knowledge, this study provides the first *in‑vivo* evidence that D_2_R availability, glucose metabolism, and FC are jointly altered in autistic adults. We found substantial increases in glucose metabolism in the ASD group, along with a modest increase in thalamic D_2_R availability in ASD. Across groups, D_2_R availability was positively associated with glucose metabolism in thalamic and striatal nuclei, indicating a tight coupling between dopaminergic function and neuronal energy demand. Importantly, higher thalamic D_2_R availability in ASD was also linked to reduced corticostriatal connectivity, demonstrating that dopaminergic alterations extend beyond local receptor changes to influence large‑scale network organization.

### D2R availability

Although limited in extent, enhanced D_2_R availability was present in the overall group comparison and more pronounced when stratified by sex, suggesting a shared dopaminergic alteration that may relate to the clinical efficacy of FDA‑approved D_2_R‑modulating agents for irritability and agitation in ASD [4]. However, this contrasts with a prior [^11^C]FLB457 PET study reporting reduced thalamic D2R BPnd in autistic males [11] and a [^11^C]raclopride PET study reporting reduced D_2_R availability in autistic males in the Nacc and GP [12]. Differences in tracer properties, imaging methodology, and sample composition may account for the discrepancy across studies. The high affinity [^11^C]FLB457 tracer is optimized for extrastriatal D_2_R measurement, whereas [^11^C]raclopride, with its moderate affinity, is the standard striatal tracer and can also reliably assess thalamic D_2_R BPnd [17, 26]. Our use of a single integrated PET/MR system with identical acquisition and reconstruction protocols across participants may further reduce inter-subject variability related to scanner characteristics, registration error, and motion compared to earlier studies. These methodological distinctions, together with difference in sample size, sex composition, and known heterogeneity in ASD phenotypes, may help explain why the prior studies reported reduced D_2_R availability [11, 12], whereas we observed elevated thalamic and striatal D_2_R availability in autistic males relative to NT males (Fig. 1E). Our findings are however consistent with a recent preclinical study comparing several male ASD mouse models and reported elevated D_2_R availability in the Nacc as a shared neurobiological feature [10]. Our findings extend this work by demonstrating sex-specific patterns of D_2_R alterations in humans. Although both autistic males and females showed increased thalamic D_2_R availability, the specific thalamic subregions involved differed by sex (Supplementary Fig. S5). These sex-dependent patterns may reflect engagement of distinct thalamocortical circuits, potentially underlying previously reported sex differences in both sensory processing [35] and thalamocortical FC in ASD [31]. This sex-specific spatial variability likely contributed to the modest overall group effect (ASD > NT) in thalamic D_2_R availability (Fig. 1A), which became more pronounced upon stratification by sex (Fig. 1D-E).

### Glucose metabolism

We observed increased glucose metabolism in the thalamus and GP of autistic individuals compared to NTs, consistent with prior reports of subcortical hypermetabolism in ASD [36]. Notably, whereas earlier [^18^F]FDG investigations focused almost exclusively on males, our data demonstrate that both autistic females and males exhibit increased bilateral thalamic and GP glucose metabolism relative to their NT counterpart (Fig. 2B,C). Together, these findings suggest that increased subcortical glucose metabolism may represent a core neurobiological marker of ASD. In alignment with this, we observed significant correlations between thalamic and GP glucose metabolism and difficulties in social skills and communication domains (Fig. 3). To our knowledge, this is the first study to demonstrate a correlation between subcortical glucose metabolism and autistic traits. However, prior fMRI studies have reported positive correlations between thalamic FC and AQ scores in ASD [37]. Together, these findings suggest that increased subcortical metabolic activity and functional coupling may scale with the degree of autistic traits.

### D2R and glucose metabolism

The voxelwise correlation analyses revealed a positive association between D_2_R availability and glucose metabolism across the bilateral caudate, GP, Nacc, thalamus, and putamen. Importantly, this relationship was observed not only in the combined sample but also when examined separately within the ASD and NT groups, indicating that the coupling between D_2_R availability and glucose metabolism represents a shared subcortical feature rather than a diagnostic artifact. Prior studies have demonstrated a positive correlation between D_2_R availability and both glucose metabolism [38] and cerebral blood flow [39], consistent with the regulatory role of dopamine in subcortical metabolic processes. Although causality cannot be inferred, the results indicate that D_2_R availability influence subcortical glucose metabolism via shared dopaminergic mechanisms in both ASD and NT groups.

### FMRI

The observed negative correlation between thalamic D_2_R availability and FC between the caudate and cortical regions (Fig. 4B) in autistic individuals suggests that elevated D_2_R availability may alter cortico-striatal and thalamo-striatal connectivity in ASD, consistent with prior fMRI findings [37, 40]. Notably, D_2_R-FC coupling differed between autistic males and females (Fig. 4D), but not among NT males and females, indicating sex-specific effects on neural communication only in the ASD group. Although both autistic males and females showed elevated D_2_R availability (Fig. 1C,E) and glucose metabolism (Fig. 2B,C), the connectivity impact appears to diverge by sex, potentially contributing to the male-biased prevalence of ASD. Prior studies in ASD also report sex differences in FC [19, 37] and exhibit that genetic risk influence FC differently across sexes [32]. Our results highlight the possibility that sex-dependent D_2_R distribution contributes to the observed discrepancies in connectivity and may underlie sex differences in ASD prevalence and clinical presentation [18].

### Limitations

Several methodological considerations should be noted. First, participation in the dual-tracer PET protocol required a high level of compliance, likely restricted enrolment to high-functioning autistic individuals. The low prevalence of psychiatric comorbidities and psychotropic medication use may further limit the generalizability to the broader ASD population. Second, [^11^C]raclopride BPnd reflects D_2_R availability but is influenced by endogenous dopamine, D3R density and receptor affinity [17, 26]. However, without pharmacological manipulation, [^11^C]raclopride BPnd is considered a reliable proxy for D_2_R density. Third, diagnostic procedures varied across clinical centres, with some centres transitioning from using the Autism Diagnostic Observation Schedule (ADOS) to the Autism Diagnostic Interview–Revised (ADI-R). This variability in diagnostic measures prevented us from examining direct correlations between ADOS/ADI-R scores and PET outcomes. Instead, we used self-reported AQ scores to provide a comparable measure of autistic traits across all participants [20]. Fourth, a potential outlier was identified in the dataset; its impact is evaluated and discussed in the Supplementary Method. Fifth, the sample primarily included White, non-Hispanic participants, which may further limit the generalizability of the findings across diverse populations. Sixth, our balanced male–female sample enabled sex‑specific analyses but does not reflect the male‑biased prevalence [41], limiting generalizability. This approach was nevertheless essential to improve representation of autistic females in neuroimaging research. Lastly, injected mass of [^11^C]raclopride could not be retrieved for all participants. All scans were performed using high specific activity tracer under standardized conditions, and injected radioactivity did not differ between groups (Table 1), suggesting that systematic differences in mass dosing are unlikely.

## Conclusion

In summary, this study demonstrates elevated subcortical D_2_R availability and glucose metabolism in autistic adults, independent of sex. We also show that D_2_R availability is tightly linked to glucose metabolism and that D_2_R-FC coupling is altered in ASD, both relative to NT adults and between autistic males and females. However, these findings are based on a cohort of cognitively able autistic adults and may not directly generalize to the broader, more heterogeneous ASD population, including children, individuals with intellectual disability, or those with significant co-occurring conditions. Together, the findings highlight dopaminergic mechanisms as potential biomarkers and therapeutic targets, while underscoring the need for further research to clarify D_2_R alterations across the heterogeneous autistic population and between sexes.

## Supplementary Information

Below is the link to the electronic supplementary material.

Supplementary file1 (DOCX 1747 KB)

## Data Availability

Data are not publicly available due to institutional restrictions but can be requested from the corresponding author with required approvals.
