# Supplementary material for: Subcortical dopamine D2 receptor availability and glucose metabolism in autism: a dual-tracer PET/MR study
Source: Eur J Nucl Med Mol Imaging. 2026 Jul 6;53(12):7419–30. doi: 10.1007/s00259-026-08053-4 (PMC13633290; doi:10.1007/s00259-026-08053-4)
Supplement: Supplementary file 1 [file 259_2026_8053_MOESM1_ESM.docx]

*European Journal of Nuclear Medicine and Molecular Imaging*

**Supplementary information:**

**Subcortical Dopamine D2 Receptor Availability and Glucose Metabolism in Autism: A Dual-Tracer PET/MR Study**

Laust Vind Knudsen^1^, Manouchehr Seyedi Vafaee^1^, Ziba Ahangarani Farahani^2^, Abigail Jane Sheldrick-Michel^1^, Tanja Maria Michel^1^
^1^ Psychiatric Research unit, Odense University Hospital, Odense, Denmark; University of Southern Denmark, Odense, Denmark
^2^ Department of Nuclear Medicine, Odense University Hospital, 5000, Odense, Denmark

**Corresponding author**
Laust Vind Knudsen, PhD. Email: [lvk@sdu.dk](mailto:lvk@sdu.dk)

**CONTENTS
Supplementary Methods
Supplementary References
Supplementary Figure S1.** Cerebellum Grey Matter as [^18^F]FDG Reference Region **Supplementary Figure S2.** Consort Diagram **Supplementary Table S1.** [^11^C]raclopride and [^18^F]FDG in ASD and NT **Supplementary Table S2.** [^11^C]raclopride and [^18^F]FDG Sex Differences

**Supplementary Figure S3.** [^11^C]raclopride BPnd Scatter Plot

**Supplementary Figure S4.** [^18^F]FDG-SUVR Scatter Plot **Supplementary Table S3.** Seed-to-Voxel Analysis Cluster Statistics  **Supplementary Figure S5.** [^11^C]raclopride Sex Differences Overlap

The authors have provided this supplementary material to offer readers additional context and details related to their study.

**Supplementary Methods

Imaging Acquisition**
All participants were scanned using a 3.0 tesla GE Healthcare SIGNA PET/MR system equipped with a 19-channel head and neck coil.

**PET Imaging**

PET imaging included both [¹¹C]raclopride and [¹⁸F]FDG scans. All PET scans were reconstructed using Bayesian penalized-likelihood iterative reconstruction (Q.Clear; β = 400), which provides improved image quality compared to ordered-subset expectation maximization [1]. Attenuation correction was performed using GE’s zero-echo-time (ZTE)-based method, optimized for brain PET/MRI. For the [¹¹C]raclopride scans, participants received a mean intravenous injection of 425.98 ± 47.55 megabecquerel (MBq), followed by a 60-minute list-mode acquisition. Image reconstruction included 32 temporal frames (1 × 105 s, 8 × 15 s, 9 × 20 s, 5 × 60 s, 1 × 195 s, and 9 × 300 s), with a voxel size of 2.34 × 2.34 × 2.78 mm³ (128 × 128 matrix; 89 slices).

For [¹⁸F]FDG PET imaging, participants were administered a mean of 303.32 ± 48.41 MBq intravenously after an overnight fast. The mean blood glucose level at the time of injection was 5.02 ± 0.41 mmol/L. The PET acquisition was initiated simultaneously with tracer injection, and a 60-minute list-mode scan was acquired and reconstructed into 37 temporal frames (12 × 5 s, 6 × 10 s, 3 × 20 s, 4 × 30 s, 5 × 60 s, 4 × 5 min, and 3 × 10 min). The voxel size and reconstruction parameters matched those used for the [¹¹C]raclopride scans (2.34 × 2.34 × 2.78 mm³; 128 × 128 matrix; 89 slices).

**MRI Imaging**

MRI acquisition included both structural and functional scans. Structural imaging used T₁-weighted 3D fast spoiled gradient-recalled echo (FSPGR BRAVO) with an echo time (TE) of 2.3 ms, repetition time (TR) of 6.5 ms, and flip angle of 12°. The matrix size was 256 × 256 (acquired) and 512 × 512 (reconstructed), with a voxel size of 0.5 × 0.5 × 0.5 mm³. Additionally, T₂-weighted 3D sagittal T₂ Cube imaging was acquired with a TE of 130 ms, TR of 6.8 s, and flip angle of 90°. The matrix size and voxel dimensions matched the T₁-weighted sequence.

Functional MRI (fMRI) was acquired using gradient echo-planar imaging (GRE-EPI) with a TE of 15 ms and TR of 2000 ms. The matrix was 64 × 64 (acquired) and 80 × 80 (reconstructed), with a voxel size of 3 × 3 × 3 mm³ and a total acquisition time of 324 seconds.

**PET Preprocessing and Analysis**
PET data were processed using the MAGIA pipeline [2] (https://github.com/tkkarjal/magia) implemented in MATLAB (The MathWorks, Inc; Natick, Massachusetts). Motion correction and image registration were conducted using Statistical Parametric Mapping (SPM12; Wellcome Centre for Human Neuroimaging, University College London; https://www.fil.ion.ucl.ac.uk/spm/software/spm12). Automated delineation of regions of interest (ROIs) was performed with FreeSurfer (version 7.3.2; https://freesurfer.net), using an optimized recon‐all procedure that incorporated both T_1_- and T_2_-weighted images to enhance registration accuracy and segmentation precision.

Ten unilateral ROIs were identified for PET analysis, including the caudate, nucleus accumbens (Nacc), globus pallidus, putamen, and thalamus, based on their previously established reliability in [¹¹C]raclopride binding potential studies [3,4]. Reference tissue volumes were automatically adjusted to account for radioligand-specific binding—utilizing the cerebellar grey matter for [^11^C]raclopride non-displaceable binding potential (BPnd) and to normalize [^18^F]-FDG uptake, thereby obtaining standardized uptake value ratios (SUVR). For [¹⁸F]FDG, SUVR images were computed using data from the 30-60 minutes post-injection interval. Cerebellar grey matter was selected as the reference region based on its common use in PET studies and was further validated in our dataset, as no group differences in cerebellar uptake were observed (Supplementary Fig. S1). Further methodological details concerning the MAGIA pipeline are available in Karjalainen et al. [2] [^11^C]raclopride BPnd was quantified using the simplified reference tissue model (SRTM) [5].

Parametric BPnd images were computed for voxelwise analysis using a basis function implementation of SRTM (bfSRTM) with 300 basis functions. Prior to parametric image generation, dynamic PET images were smoothed with a 4-mm full-width at half-maximum (FWHM) Gaussian kernel to reduce noise. The resulting parametric images were normalized to MNI152 space and subsequently smoothed with an additional 4-mm Gaussian filter. An identical procedure was applied to generate [¹⁸F]FDG parametric images. Parametric [¹¹C]raclopride BPnd and [¹⁸F]FDG SUVR images were extracted from MAGIA and merged into a 4D image containing one volume per participant (N = 60). Group comparisons between individuals with autism spectrum disorder (ASD) and neurotypical (NT) controls were conducted using FSL’s randomise tool with threshold-free cluster enhancement (TFCE) [6]. The [¹¹C]raclopride analysis controlled for age and sex, while the [¹⁸F]FDG analysis additionally adjusted for body mass index (BMI). Both analyses were restricted to a mask comprising the bilateral caudate, Nacc, globus pallidus, putamen, and thalamus, derived from the Harvard-Oxford Subcortical Structural Atlas [7]. This mask was thresholded at 50% probability, binarized, and included 5307 voxels with a total volume of 42,456 mm³.

Moreover, Voxelstats [8] was used to assess voxelwise correlations between [¹¹C]raclopride BPnd and [^18^F]FDG SUVR, adjusting for age and sex using the model: Nifti_Rac∼Nifti_FDG+Age+Sex. Cluster-level multiple comparisons were corrected for using random field theory [9].

**fMRI Preprocessing**
Functional MRI data from 60 participants were preprocessed and analyzed using the CONN functional connectivity toolbox (version 22.a) implemented in MATLAB R2021b (MathWorks Inc) [10]. Preprocessing followed CONN’s default pipeline, which included realignment and unwarping, slice-timing correction, segmentation and normalization to Montreal Neurological Institute (MNI) space, co-registration to the structural reference image, detection of outlier volumes, and spatial smoothing with an 8-mm full-width at half-maximum Gaussian kernel. Physiological and motion-related noise components were removed using component-based noise correction (CompCor), and data were bandpass filtered at 0.008–0.09 Hz. Motion artifacts were identified using the Artifact Detection Toolbox (ART), with outlier volumes defined as exceeding the 97th percentile of motion estimates. Volumes were scrubbed if they exhibited a framewise displacement ≥ 0.9 mm or a blood oxygen level–dependent (BOLD) signal deviation ≥ ±5 standard deviations from the mean global signal.

**fMRI Analysis**

Multivoxel pattern analysis (MVPA), as implemented in the CONN toolbox, was used as a data-driven approach to identify seed regions for subsequent seed-to-voxel functional connectivity (FC) analyses. This study aimed to identify regions where whole-brain FC exhibited a significant interaction between dopamine D2 receptor (D2R) availability and diagnostic group. Given that [^11^C]raclopride BPnd in the left thalamus differed significantly between the autistic and NT group, this measure was used as the index of D2R availability. MVPA was conducted by computing multivariate FC patterns between each voxel and all other brain voxels. Dimensionality reduction was performed using principal component analysis (PCA), retaining the first five components, which regularly accounts for approximately 80% to 85% of whole-brain FC variance [11].
Group-level effects were assessed using multivariate analysis of covariance (MANCOVA), with four independent variables: group membership (ASD = 1, NT = 0 and vice versa) and individual group-specific [^11^C]raclopride BPnd values. The contrast vector [0 0 1 −1] (i.e. ASD, NT, BPnd_ASD, BPnd_NT) was used to test for between-group differences in the association between FC patterns and D2R availability. Statistical maps were masked to gray matter and thresholded at p < .001 (voxelwise, uncorrected, two-tailed) and p < .05 (cluster-level, false discovery rate–corrected). The same procedure was applied to test for differences between ASD males and females. In post hoc analyses, the significant MVPA clusters were used as seed regions in seed-to-voxel FC analyses to further characterize group differences in brain-wide connectivity. The same thresholding criteria were applied.

**Statistical ROI Analysis**

Statistical analyses of region of interest (ROI) data from MAGIA were conducted using R (version 2024.12.1+563; R Foundation for Statistical Computing). Data were imported from comma-separated value (CSV) files. Categorical variables were recoded as factors: sex was coded as 1 (male) and 0 (female), and diagnostic group as 1 (ASD and 0 NT). Ten bilateral subcortical regions were analyzed as dependent variables: left and right caudate, Nacc, putamen, thalamus, and globus pallidus. To examine group differences in [^11^C]raclopride BPnd, age and sex were included as covariates. For the analysis of [^18^F]FDG SUVR, age, sex, and body mass index were included as covariates. Multivariate group effects were assessed using a permutation-based analysis of covariance (ANCOVA), implemented with the aovp function from the *lmPerm* package. Pillai’s trace was used as the omnibus test statistic, given its robustness to violations of normality and homogeneity of variance.

Follow-up univariate ANCOVAs were conducted within sex subgroups to explore diagnostic group differences. Separate models were estimated for male and female participants, comparing ASD and NT groups across each ROI. All univariate models were conducted using permutation-based ANCOVA to maintain consistency with the multivariate approach. Effect sizes were estimated using partial eta-squared (ηp²), computed via the etaSquared function from the *lsr* package. P values from the univariate tests were corrected for multiple comparisons using the false discovery rate (FDR) method, applied within each family of hypotheses [12]. This analytic approach enabled both a global multivariate assessment of diagnostic, demographic, and biological effects on BPnd, as well as targeted post hoc testing to identify region-specific differences between ASD and NT participants within each sex group, while controlling for key confounding variables.

**Correlation Analysis**
To examine associations between Autism-Spectrum Quotient (AQ) scores and regional PET measures, Spearman rank correlation coefficients (ρ) were computed. Spearman correlation was selected due to its robustness to non-normal distributions and its capacity to detect monotonic relationships. For each AQ score and brain region pair, correlation analyses were conducted using the *cor.test* function in R with the method set to “spearman,” restricting analyses to complete cases.

Because PET measures may be influenced by age and sex, a permutation-based ANCOVA was used to test the association between AQ scores and regional PET measures while adjusting for these covariates. Specifically, for each brain region and AQ score pair, models of the form: Brain Region ~ AQ Score + Age + Sex were estimated using the *lmPerm* package. The permutation-based ANCOVA approach was configured to run with a maximum of 20,000 iterations and a convergence criterion of 0.01, allowing for nonparametric estimation of p values. Resulting p values were adjusted for multiple comparisons using the FDR method.

To visualize results, heatmaps were generated using the *ggplot2* package, representing Spearman correlation coefficients alongside FDR-adjusted p values. Each heatmap cell displayed the correlation coefficient (rounded to two decimal places) and corresponding p value (rounded to three decimal places); statistically significant results (FDR-adjusted p < .05) were displayed in bold.

**Clinical Assessment**
We selected the Autism Spectrum Quotient (AQ) as the primary measure of autistic traits, as it was completed by all 60 participants. Due to changes in clinical diagnostic protocols at our recruitment site in Odense, not all autistic participants were assessed using the same tool. Eighteen underwent the Autism Diagnostic Observation Schedule (ADOS), with mean (SD) scores of 10.27 (2.83) for Social Affect, 0.82 (1.08) for Restricted and Repetitive Behavior (RRB), and 10.75 (3.36) total. Twelve were assessed with the Autism Diagnostic Interview–Revised (ADI-R), showing mean (SD) scores of 12.00 (3.80) for Social Interaction, 10.11 (2.67) for Verbal Behavior, and 3.44 (0.73) for RRB. One participant completed the Ritvo Autism Asperger Diagnostic Scale–Revised (RAADS; score = 94), one the Autism Spectrum Screening Questionnaire (ASSQ; score = 38), and one the Social Responsiveness Scale (SRS; score = 88).

**Participants**
Adults (age ≥ 20 years) with full‐scale IQ > 80 and capacity to consent were enrolled in this case–control study. Autistic participants (n = 30) were referred by the diagnostic team in Odense or recruited via advertisements; all held a prior clinical diagnosis of ASD according to DSM-5 criteria [13]. We aimed to recruit individuals not taking medication and without psychiatric or neurological comorbidities to minimize potential confounding. However, five ASD participants were on stable medications: two were taking antidepressants (sertraline, venlafaxine) and paused these ≥48 hours before scanning; the remaining three continued levothyroxine (hypothyroidism), mesalazine (ulcerative colitis), or nightly melatonin (1 mg for sleep disturbances). NT participants (n = 30) were recruited via public advertisement. Recruitment was stratified to achieve a balanced 1:1 male-to-female ratio within each diagnostic group, ensuring adequate representation of females and enabling investigation of sex effects despite the male-biased prevalence of ASD [14].

All participants completed the 50-item Autism-Spectrum Quotient (AQ) to quantify autistic traits [15] and the Edinburgh Handedness Inventory [16]. Cognitive ability was determined from existing Wechsler Adult Intelligence Scale (WAIS) or Wechsler Intelligence Scale for Children (WISC) scores; those without prior assessment in their medical record completed a shortened WAIS-IV (Supplementary Methods). Exclusion criteria for both groups included claustrophobia or metal implants contraindicating MRI, brain abnormalities on structural MRI scans (as assessed by an experienced physician), any neurological diagnosis, use of nicotine or psychostimulants, and failure to fast overnight (verified by blood glucose measurement and defined as abstaining from all caloric and caffeine intake, as caffeine can influence both D2R availability [17] and cerebral metabolism [18]).

**Cognitive Assessment**
Following signed participant consent, we were able to assess autistic participants medical journal, to assess the diagnosis including if a measure of IQ were assessed using Wechsler Adult Intelligence Scale (WAIS) or Wechsler Intelligence Scale for Children (WISC). Participants who were missing an intelligence test, or where the test score were missing, was asked to perform a short-form of the WAIS-IV ad our facilities. Additionally, all neurotypical individuals were asked to perform this test.
To estimate Full-Scale IQ (FSIQ), we employed a short-form version of the WAIS-IV consisting of four subtests: Digit Span, Matrix Reasoning, Vocabulary, and Symbol Search. This approach was informed by prior research demonstrating a high concordance between abbreviated WAIS-IV assessments and FSIQ scores, with mean score discrepancies as small as 0.18 IQ points [19]. Drawing on the methodology described by Tellegen and Briggs [20] and operationalized in Tables B-10 and B-11 of Sattler and Ryan [21], we derived FSIQ estimates using a linear scaling procedure. This method preserves the distributional properties of the full-scale IQ (mean = 100; standard deviation = 15) and has been validated in prior evaluations of short-form accuracy [19,22]. This procedure was used to estimate IQ for neurotypical individuals, as well as for autistic participants for whom a full WAIS-IV assessment was not available. In total, 28 neurotypical individuals and six autistic individuals completed the short-form WAIS. Two neurotypical individuals did not undergo cognitive assessment.

**Potential Participant Exclusion**
Female participants were asked and encouraged to verify whether they were pregnant or might be pregnant at the time of scanning. One participant, who had taken a home pregnancy test on the morning of the scan and received a negative result, was weeks later found to be in the very early stages of pregnancy. A hospital physicist calculated the estimated total radiation exposure from the scan to be approximately 8 millisieverts, which was evaluated as not posing a significant risk to the fetus at this low level of exposure.

We considered excluding this participant’s data from analysis and therefore evaluated whether her PET measurements deviated meaningfully from those of the other female autistic participants. Her [¹¹C]raclopride BPnd values diverged by 16.44% from the mean of the other 14 autistic females. Across the female ASD group, individual [¹¹C]raclopride BPnd deviations from the group mean ranged from 3.40% to 32.90%. Similarly, the participant’s [¹⁸F]FDG SUVR differed by 4.21%, while the absolute SUVR deviations from the mean among the other female ASD participants ranged from 2.96% to 28.75%. Based on these findings, her PET measures were not considered outliers within the female ASD group.

Given the negative pregnancy test and participant-reported information, it is expected that the scan took place within the first ~3 weeks of pregnancy, a stage characterized by low levels of human chorionic gonadotropin (hCG). Estradiol levels increase during pregnancy [23,24], but significant elevations occur later in pregnancy [24]. While preclinical studies suggest that estradiol can reduce dopamine D2R availability in rodents [25], human studies have not consistently demonstrated similar effects related to menstrual cycle-induced fluctuations in estradiol [26,27]. Furthermore, estradiol levels during early pregnancy resemble those observed during the menstrual cycle [23,27], supporting the notion that any influence on D2R availability would likely be minimal at this early stage. Considering all the above, we decided not to exclude this participant’s data from the study.

**Supplementary References**

1. Teoh EJ, McGowan DR, Macpherson RE, Bradley KM, Gleeson F V. Phantom and clinical evaluation of the Bayesian penalized likelihood reconstruction algorithm Q.Clear on an LYSO PET/CT system. Journal of Nuclear Medicine. 2015;56:1447–52. https://doi.org/10.2967/jnumed.115.159301

2. Karjalainen T, Tuisku J, Santavirta S, Kantonen T, Bucci M, Tuominen L, et al. Magia: Robust Automated Image Processing and Kinetic Modeling Toolbox for PET Neuroinformatics. Front Neuroinform. Frontiers Media S.A.; 2020;14:1–13. https://doi.org/10.3389/fninf.2020.00003

3. Malén T, Karjalainen T, Isojärvi J, Vehtari A, Bürkner PC, Putkinen V, et al. Atlas of type 2 dopamine receptors in the human brain: Age and sex dependent variability in a large PET cohort. Neuroimage. Academic Press Inc.; 2022;255:1–9. https://doi.org/10.1016/j.neuroimage.2022.119149

4. Spagnolo PA, Kimes A, Schwandt ML, Shokri-Kojori E, Thada S, Phillips KA, et al. Striatal Dopamine Release in Response to Morphine: A [11C]Raclopride Positron Emission Tomography Study in Healthy Men. Biol Psychiatry. Elsevier USA; 2019;86:356–64. https://doi.org/10.1016/j.biopsych.2019.03.965

5. Lammertsma AA, Hume SP. Simplified Reference Tissue Model for PET Receptor Studies. Neuroimage. 1996;4:153–8. https://doi.org/10.1006/nimg.1996.0066

6. Winkler AM, Ridgway GR, Webster MA, Smith SM, Nichols TE. Permutation inference for the general linear model. Neuroimage [Internet]. The Authors; 2014;92:381–97. https://doi.org/10.1016/j.neuroimage.2014.01.060

7. Desikan RS, Ségonne F, Fischl B, Quinn BT, Dickerson BC, Blacker D, et al. An automated labeling system for subdividing the human cerebral cortex on MRI scans into gyral based regions of interest. Neuroimage. 2006;31:968–80. https://doi.org/10.1016/j.neuroimage.2006.01.021

8. Mathotaarachchi S, Wang S, Shin M, Pascoal TA, Benedet AL, Kang MS, et al. VoxelStats: A MATLAB package for multi-modal voxel-wise brain image analysis. Front Neuroinform. 2016;10:1–12. https://doi.org/10.3389/fninf.2016.00020

9. Worsley KJ, Marrett S, Neelin P, Vandal AC, Friston KJ, Evans AC. A unified statistical approach for determining significant signals in images of cerebral activation. Hum Brain Mapp. John Wiley and Sons Inc; 1996;4:58–73. https://doi.org/10.1002/(SICI)1097-0193(1996)4:1<58::AID-HBM4>3.0.CO;2-O

10. Nieto-Castanon A, Whitfield-Gabrieli S. CONN functional connectivity toolbox: RRID SCR_009550, release 22. Boston: Hilbert Press Manual / Technical Report Library; 2022. p. 1–62. https://doi.org/10.56441/hilbertpress.2246.5840

11. Wang Y, Bernanke J, Peterson BS, McGrath P, Stewart J, Chen Y, et al. The association between antidepressant treatment and brain connectivity in two double-blind, placebo-controlled clinical trials: a treatment mechanism study. Lancet Psychiatry. Elsevier Ltd; 2019;6:667–74. https://doi.org/10.1016/S2215-0366(19)30179-8

12. Benjamini Y, Hochberg Y. Controlling the False Discovery Rate: A Practical and Powerful Approach to Multiple. Journal of the Royal Statistical Society. 1995;57:289–300. https://doi.org/10.1111/j.2517-6161.1995.tb02031.x

13. American Psychiatric Association. Diagnostic and Statistical Manual of Mental Disorders (DSM-5). American Psychiatric Pub. 2013;1121:10–32. https://doi.org/10.1196/annals.1401.015

14. Shaw KA, Williams S, Patrick ME, Valencia-Prado M, Maureen SD, Howerton EllenM, et al. Prevalence and Early Identification of Autism Spectrum Disorder Among Children Aged 4 and 8 Years-Autism and Developmental Disabilities Monitoring Network, 16 Sites, United States, 2022. Health and Human Services | Centers for Disease Control and Prevention | MMWR. 2025;74:1–22.

15. Baron-Cohen S, Wheelwright S, Skinner R, Martin J, Clubley E. The Autism-Spectrum Quotient (AQ): Evidence from Asperger Syndrome/High-Functioning Autism, Males and Females, Scientists and Mathematicians. J Autism Dev Disord. 2001;31:5–17. https://doi.org/10.1023/a:1005653411471.

16. Oldfield RC. The Assessment and Analysis of Handedness: The Edinburgh Inventory. Neuropsychologia. Pergamon Press; 1971;9:97–113.

17. Volkow ND, Wang GJ, Logan J, Alexoff D, Fowler JS, Thanos PK, et al. Caffeine increases striatal dopamine D2/D3 receptor availability in the human brain. Transl Psychiatry. Nature Publishing Group; 2015;5. https://doi.org/10.1038/tp.2015.46

18. Vidyasagar R, Greyling A, Draijer R, Corfield DR, Parkes LM. The effect of black tea and caffeine on regional cerebral blood flow measured with arterial spin labeling. Journal of Cerebral Blood Flow and Metabolism [Internet]. Nature Publishing Group; 2013;33:963–8. https://doi.org/10.1038/jcbfm.2013.40

19. van Ool JS, Hurks PPM, Snoeijen-Schouwenaars FM, Tan IY, Schelhaas HJ, Klinkenberg S, et al. Accuracy of WISC-III and WAIS-IV short forms in patients with neurological disorders. Dev Neurorehabil. Taylor and Francis Ltd.; 2018;21:101–7. https://doi.org/10.1080/17518423.2016.1277799

20. Tellegen A, Briggs PF. old wine in new skins: grouping wechsler subtests into new scales 1. Journal oj Consulting Psychology. 1967.

21. Knudsen L V., Sheldrick AJ, Vafaee MS, Michel TM. Diversifying autism neuroimaging research: An arterial spin labeling review. Autism. 2023;27:1190–203. https://doi.org/10.1177/13623613221137230

22. Girard TA, Axelrod BN, Patel R, Crawford JR. Wechsler Adult Intelligence Scale–IV Dyads for Estimating Global Intelligence. Assessment. SAGE Publications Inc.; 2015;22:441–8. https://doi.org/10.1177/1073191114551551

23. Boyle P, Andralojc K, van der Velden S, Najmabadi S, de Groot T, Turczynski C, et al. Restoration of serum estradiol and reduced incidence of miscarriage in patients with low serum estradiol during pregnancy: a retrospective cohort study using a multifactorial protocol including DHEA. Frontiers in Reproductive Health. Frontiers Media SA; 2023;5. https://doi.org/10.3389/frph.2023.1321284

24. Chapman AB, Abraham WT, Zamudio S, Coffin C, Merouani A, Young D, et al. CLINICAL NEPHROLOGY-EPIDEMIOLOGY-CLINICAL TRIALS Temporal relationships between hormonal and hemodynamic changes in early human pregnancy. 1998.

25. Bazzett TJ, Becket JB. Sex differences in the rapid and acute effects of estrogen on striatal O 2 dopamine receptor binding. 1994.

26. Nordstrom A-L, Olsson H, Halldin¨ H. A PET study of D dopamine receptor density at different 2 phases of the menstrual cycle. Psychiatry Research: Neuroimaging Section. 1998.

27. Petersen N, Rapkin AJ, Okita K, Kinney KR, Mizuno T, Mandelkern MA, et al. Striatal dopamine D2-type receptor availability and peripheral 17β-estradiol. Mol Psychiatry. Springer Nature; 2021;26:2038–47. https://doi.org/10.1038/s41380-020-01000-1

**Supplementary Figure S1. Cerebellum Grey Matter as [^18^F]FDG Reference Region**

To confirm that the cerebellar gray matter did not introduce bias in the SUVR analysis, we examined the time-activity curves of all participants and compared uptake between groups. The NT group showed a mean cerebellar uptake of 33,548 ± 4,577 kBq/mL, while the ASD group showed 33,130 ± 5,608 kBq/mL. A two-sample t-test indicated no significant group difference, t(58) = t = −0.31, p = 0.753, d = −0.08 [95% CI −0.58, 0.42], supporting the use of the cerebellum as an appropriate reference region.

**Supplementary Figure S2. Consort Diagram**. The flow diagram describes the cause for exclusion or dropout of the study.

PET = Positron emission tomography. n = number.

**Supplementary Table S1. [^11^C]raclopride and [^18^F]FDG in ASD and NT**.

| **Brain region** | **ASD (N=30)** | | | **NT (N=30)** | | | **Analysis** | | |
| --- | --- | --- | --- | --- | --- | --- | --- | --- | --- |
|  | Mean | SD | | Mean | SD | p | | p-FDR | ηp² |
|  | **[^11^C]raclopride BPnd** | | | | | | | | |
| Caudate L | 2.75 | 0.37 | 2.68 | | 0.24 | 0.861 | | 0.990 | <0.001 |
| Caudate R | 2.98 | 0.42 | 2.93 | | 0.23 | 0.983 | | 0.990 | <0.001 |
| Nacc L | 2.33 | 0.32 | 2.23 | | 0.27 | 0.110 | | 0.367 | 0.043 |
| Nacc R | 1.65 | 0.35 | 1.65 | | 0.32 | 0.878 | | 0.990 | <0.001 |
| Putamen L | 3.65 | 0.51 | 3.64 | | 0.33 | 0.703 | | 0.990 | 0.003 |
| Putamen R | 3.58 | 0.50 | 3.51 | | 0.32 | 0.990 | | 0.990 | <0.001 |
| Thalamus L | 0.35 | 0.09 | 0.32 | | 0.04 | 0.045 | | 0.367 | 0.057 |
| Thalamus R | 0.34 | 0.09 | 0.31 | | 0.04 | 0.074 | | 0.367 | 0.052 |
| GP L | 1.43 | 0.24 | 1.34 | | 0.26 | 0.159 | | 0.398 | 0.035 |
| GP R | 0.95 | 0.19 | 0.88 | | 0.14 | 0.357 | | 0.714 | 0.017 |
|  | **[^18^F]FDG SUVR** | | | | | | | | |
| Caudate L | 1.02 | 0.14 | 0.99 | | 0.08 | 0.491 | | 0.491 | 0.007 |
| Caudate R | 1.05 | 0.14 | 1.01 | | 0.08 | 0.256 | | 0.284 | 0.023 |
| Nacc L | 1.06 | 0.12 | 1.02 | | 0.09 | 0.038 | | 0.088 | 0.074 |
| Nacc R | 0.99 | 0.11 | 0.97 | | 0.09 | 0.231 | | 0.284 | 0.030 |
| Putamen L | 1.24 | 0.13 | 1.19 | | 0.09 | 0.209 | | 0.284 | 0.028 |
| Putamen R | 1.22 | 0.12 | 1.18 | | 0.09 | 0.138 | | 0.272 | 0.037 |
| Thalamus L | 0.94 | 0.11 | 0.89 | | 0.06 | 0.009 | | 0.047 | 0.111 |
| Thalamus R | 0.96 | 0.11 | 0.93 | | 0.06 | 0.024 | | 0.047 | 0.079 |
| GP L | 0.83 | 0.09 | 0.80 | | 0.08 | 0.163 | | 0.272 | 0.039 |
| GP R | 0.84 | 0.09 | 0.80 | | 0.07 | 0.010 | | 0.047 | 0.110 |
|  |  |  |  | |  |  | |  |  |

ASD = autism spectrum disorder. ηp² = partial eta squared. FDR = false discovery rate. GP = Globus Pallidus. L = left. Nacc = nucleus accumbens. NT = neurotypical. R = right.

**Supplementary Table S2. [^11^C]raclopride and [^18^F]FDG Sex Differences**

| **Brain region** | ***ASD M vs. F*** | | ***NT M vs. F*** | | ***NT F vs. ASD F*** | | **NT M vs ASD M** | |
| --- | --- | --- | --- | --- | --- | --- | --- | --- |
|  | p | ηp² | p | ηp² | p | ηp² | p | ηp² |
|  | **[^11^C]raclopride BPnd** | | | | | | | |
| Caudate L | 0.128 | 0.141 | 0.485 | 0.108 | 0.757 | 0.015 | 0.950 | 0.003 |
| Caudate R | 0.128 | 0.138 | 0.485 | 0.076 | 0.757 | 0.016 | 0.950 | 0.016 |
| Nacc L | 0.464 | 0.022 | 0.485 | 0.078 | 0.757 | 0.017 | 0.470 | 0.144 |
| Nacc R | 0.316 | 0.059 | 0.636 | 0.022 | 0.757 | 0.011 | 0.950 | 0.016 |
| Putamen L | 0.396 | 0.032 | 0.485 | 0.065 | 0.757 | 0.003 | 0.950 | 0.003 |
| Putamen R | 0.316 | 0.055 | 0.636 | 0.018 | 0.757 | 0.004 | 0.950 | <0.001 |
| Thalamus L | 0.055 | 0.169 | 0.636 | 0.032 | 0.130 | 0.132 | 0.950 | <0.001 |
| Thalamus R | 0.055 | 0.176 | 0.637 | 0.014 | 0.130 | 0.137 | 0.950 | 0.013 |
|  | **[^18^F]FDG SUVR** | | | | | | | |
| Caudate L | 0.027 | 0.236 | 0.795 | 0.080 | 0.192 | 0.067 | 0.728 | 0.029 |
| Caudate R | 0.027 | 0.235 | 0.473 | 0.090 | 0.170 | 0.078 | 0.838 | 0.002 |
| Nacc L | 0.161 | 0.089 | 0.473 | 0.130 | 0.168 | 0.090 | 0.695 | 0.048 |
| Nacc R | 0.047 | 0.186 | 0.473 | 0.008 | 0.260 | 0.046 | 0.728 | 0.010 |
| Putamen L | 0.149 | 0.106 | 0.883 | 0.005 | 0.170 | 0.080 | 0.728 | 0.007 |
| Putamen R | 0.149 | 0.092 | 0.883 | 0.008 | 0.168 | 0.093 | 0.728 | 0.016 |
| Thalamus L | 0.174 | 0.065 | 0.883 | 0.009 | 0.073 | 0.183 | 0.695 | 0.061 |
| Thalamus R | 0.174 | 0.067 | 0.883 | 0.023 | 0.157 | 0.123 | 0.695 | 0.044 |
|  |  |  |  |  |  |  |  |  |

P-values are false discovery rate corrected. M = male. F = female. L = left. R = right. ASD = autism spectrum disorder. BPnd = non-displaceable binding potential. ηp² = partial eta-squared. GP = globus pallidus. Nacc = nucleus accumbens. NT = neurotypical.

**Supplementary Figure S3. [^11^C]raclopride BPnd Scatter Plot.**

Groups were separated based on diagnosis and gender. L = left. R = right. ASD = autism spectrum disorder. BPnd = non-displaceable binding potential. GP = globus pallidus. Nacc = nucleus accumbens. NT = Neurotypicals.

**Supplementary Figure S4. [^18^F]FDG-SUVR Scatter Plot.**

Group were separated based on diagnosis and gender. L = left. R = right. ASD = autism spectrum disorder. GP = globus pallidus. Nacc = nucleus accumbens. NT = neurotypical. SUVR = standardized uptake value ratio.

**Supplementary Table S3. Seed-to-Voxel Analysis Cluster Statistics.**

**A)** The results are from the exploration of how left thalamic [^11^C]raclopride BPnd modulates functional connectivity in the autistic and neurotypical group from the cluster located in the left caudate. **B)** the same analysis between autistic males and females.

| Cluster (x,y,z) | size | size p-FWE | size p-FDR | size p-unc | peak p-FWE | peak p-unc | +/- |
| --- | --- | --- | --- | --- | --- | --- | --- |
| **A** | Seed to voxel from left caudate | | | | | | |
| +18 -78 +36 | 371 | 0.00001 | 0.00001 | 0.00000 | 0.44263 | 0.00001 |  |
| +46 +36 +30 | 354 | 0.00001 | 0.00001 | 0.00000 | 0.22875 | 0.00000 |  |
| +06 -30 +34 | 245 | 0.00028 | 0.00023 | 0.00001 | 0.95445 | 0.00005 |  |
| -26 -66 +42 | 205 | 0.00103 | 0.00064 | 0.00003 | 0.71384 | 0.00002 |  |
| -66 -52 +10 | 186 | 0.00196 | 0.00098 | 0.00006 | 0.11297 | 0.00000 |  |
| +24 -70 -08 | 181 | 0.00234 | 0.00098 | 0.00007 | 0.09200 | 0.00000 |  |
| **B** | Seed to voxel from left frontal orbital cortex | | | | | | |
| -02 +38 +56 | 1492 | 0.00000 | 0.00000 | 0.00000 | 0.03683 | 0.00000 | + |
| +12 -60 +58 | 334 | 0.00000 | 0.00000 | 0.00000 | 0.67769 | 0.00001 | - |
| +22 -86 -36 | 207 | 0.00033 | 0.00027 | 0.00000 | 0.35334 | 0.00000 | + |
| -08 +58 -02 | 204 | 0.00037 | 0.00027 | 0.00001 | 0.50680 | 0.00000 | + |
| +20 +40 +34 | 174 | 0.00119 | 0.00062 | 0.00003 | 0.36930 | 0.00000 | + |
| -50 -58 +30 | 172 | 0.00129 | 0.00062 | 0.00003 | 0.89398 | 0.00002 | + |
| -60 -04 -16 | 105 | 0.02275 | 0.00957 | 0.00061 | 0.81362 | 0.00001 | + |
| -22 -52 +60 | 86 | 0.05620 | 0.02104 | 0.00154 | 0.86284 | 0.00002 | - |
| +02 -42 +36 | 79 | 0.07927 | 0.02671 | 0.00220 | 0.51577 | 0.00000 | + |
| +02 -52 -10 | 75 | 0.09671 | 0.02960 | 0.00271 | 0.93016 | 0.00002 | - |

FWE = family wise error. FDR = False discovery rate. The right most column indicates whether the correlation was positive (stronger in the male group) or negative (stronger in the female group) between left thalamic BPnd and FC from the seed.

**Supplementary Figure S4. [^11^C]raclopride Sex Differences Overlap**

The figure illustrates [¹¹C]raclopride BPnd differences between ASD and NT individuals, with ASD vs. NT males shown in red and ASD vs. NT females shown in green. The spatial distribution of these group differences suggests the presence of sex-specific mechanisms.
